# Rhythmic Diurnal Synthesis and Signaling of Retinoic Acid in the Rat Pineal Gland and Its Action to Rapidly Downregulate ERK Phosphorylation

**DOI:** 10.1007/s12035-018-0964-5

**Published:** 2018-03-08

**Authors:** Anna Ashton, Patrick N. Stoney, Jemma Ransom, Peter McCaffery

**Affiliations:** 1 Institute of Medical Sciences, School of Medicine, Medical Sciences and Nutrition, University of Aberdeen, Foresterhill, Aberdeen, Scotland AB25 2ZD UK; 2 Cell Signal Unit, Okinawa Institute of Science and Technology, Okinawa, Japan

**Keywords:** Retinoic acid, Retinol, ERK, Circadian, Pineal gland, rdh10, rdh12, RARgamma

## Abstract

Vitamin A is important for the circadian timing system; deficiency disrupts daily rhythms in activity and clock gene expression, and reduces the nocturnal peak in melatonin in the pineal gland. However, it is currently unknown how these effects are mediated. Vitamin A primarily acts via the active metabolite, retinoic acid (RA), a transcriptional regulator with emerging non-genomic activities. We investigated whether RA is subject to diurnal variation in synthesis and signaling in the rat pineal gland. Its involvement in two key molecular rhythms in this gland was also examined: kinase activation and induction of *Aanat*, which encodes the rhythm-generating melatonin synthetic enzyme. We found diurnal changes in expression of several genes required for RA signaling, including a RA receptor and synthetic enzymes. The RA-responsive gene *Cyp26a1* was found to change between day and night, suggesting diurnal changes in RA activity. This corresponded to changes in RA synthesis, suggesting rhythmic production of RA. Long-term RA treatment in vitro upregulated *Aanat* transcription, while short-term treatment had no effect. RA was also found to rapidly downregulate extracellular signal-regulated kinase (ERK) 1/2 phosphorylation, suggesting a rapid non-genomic action which may be involved in driving the molecular rhythm in ERK1/2 activation in this gland. These results demonstrate that there are diurnal changes in RA synthesis and activity in the rat pineal gland which are partially under circadian control. These may be key to the effects of vitamin A on circadian rhythms, therefore providing insight into the molecular link between this nutrient and the circadian system.

## Introduction

Vitamin A is an essential dietary component that is delivered to tissues in the form of retinol. It primarily acts through two active metabolites, synthesized in a two-step oxidative pathway. The first, retinaldehyde, is used in the visual cycle for the functioning of rhodopsin in phototransduction. Retinoic acid (RA), the metabolite of retinaldehyde, is a potent transcriptional regulator in the central nervous system (CNS) through activation of ligand-gated transcription factors, retinoic acid receptors (RARs) [1]. In addition to its canonical genomic activities, RA also has non-genomic effects including regulation of protein translation [2–4] and kinase phosphorylation; studies have demonstrated that RA modulates activation of a number of kinases including extracellular signal-regulated kinase (ERK) 1/2 and Akt [5–7].

The role of RA in embryonic development is well established [8] and it remains active in the adult CNS where it has roles in neurogenesis [9, 10] and synaptic plasticity [11, 12]. There is increasing evidence emerging for a new role of RA in the regulation of circadian rhythms (reviewed in Ransom et al. [13]). Vitamin A deficiency (VAD) has been found to disrupt oscillations in clock gene expression [14, 15] and daily rhythms of locomotor activity, a direct output of the central circadian clock [16]. Currently, it is not clear how these effects are mediated, but previous studies have alluded to a role for RA. The three isomers of RA, all-*trans*-RA, 9-*cis* RA and 13-*cis* RA, were identified as potential circadian entrainment factors in a screen of 299 peptides and bioactive lipids, due to their ability to entrain rhythmic expression of the clock gene, *Per2* [17]. RA also influences other components of the circadian clock, including inhibition of CLOCK:BMAL [18, 19], and upregulation of *Per1* [19]. Furthermore, the RAR-related orphan receptor (ROR) β, for which RA is a high-affinity ligand [20], is highly expressed in the circadian system [21, 22], and its deletion leads to an extended free-running period in mice [23, 24]. In addition, studies suggest vitamin A is necessary for pineal gland function, whose primary purpose is to regulate physiological rhythms by relaying the signal of circadian time to the body via melatonin secretion. The suprachiasmatic nucleus (SCN), the site of the master clock, drives the nocturnal increase in melatonin production by inducing activation of arylalkylamine N-acetyltransferase (AANAT) at night, the penultimate melatonin synthetic enzyme. In a number of vertebrates, including rodents, the AANAT rhythm is predominantly under transcriptional control with an increase in *Aanat* mRNA of more than a 150-fold at night in rats [25]. Studies have found that VAD in rats leads to a significant reduction in peak nocturnal AANAT activity and melatonin levels, as well as the disappearance of the daily rhythm in mitogen-activated protein kinase (MAPK) activation [26, 27]. However, it is currently unknown how these effects are mediated.

Retinol has roles in the pineal gland of non-mammalian vertebrates; in reptiles it is required for photoreceptor integrity [28] and in birds it is involved in phototransduction [29, 30]. However, its role in the mammalian pineal gland has scarcely been investigated, despite it containing high levels of retinol and retinol binding proteins [31–33]. Retinaldehyde is undetectable in the mammalian pineal gland indicating that it is not likely to serve a phototransduction role [32, 34]. Whereas genes associated with RA signaling have been reported to be highly expressed in the rodent pineal gland [35], suggesting that RA is active in the mammalian pineal gland. These include genes encoding the retinoid X receptor (RXR) γ, which binds to the 9-*cis* isomer of RA [36], and RORβ, which is rhythmically expressed in the rat pineal gland with a peak during the night [22, 37].

In order for RA to be a regulator of circadian rhythms, it should itself be subject to diurnal variations in activity. In this study, focussing on the pineal gland component of the circadian system, we determined that RA synthesis and signaling do indeed exhibit diurnal changes. The effect of RA on *Aanat* transcription and kinase activation, which comprise two key molecular rhythms in this gland, was also investigated. Several of the genes involved in RA signaling displayed diurnal changes in expression and production of RA by the pineal gland was found to increase during the night, suggesting there is a diurnal rhythm in RA synthesis and signaling in this gland. Short-term treatment of RA did not influence *Aanat* transcription in cultured pineal glands indicative that RA would not control *Aanat* rhythmicity, although long-term treatment induced a twofold upregulation in expression, suggesting that RA may influence the amplitude of AANAT’s rhythmical expression. In addition, RA induced a rapid decrease in ERK1/2 phosphorylation, demonstrating that it has the capacity to influence kinase activity and therefore molecular rhythms in the rat pineal gland. ERK1/2 signaling is subject to diurnal oscillations in activity in the pineal gland with an increase in phosphorylated levels during the night [26, 38], which likely enables its role in the regulation of the circadian pacemaker in this gland [39, 40]. In addition, it is also thought to be a key mediator of photoentrainment in the SCN [41–43]. Therefore, the phosphorylated level of ERK1/2 has an important role in biological timekeeping, and the present study supports the growing evidence that RA should also be considered as an important component of the circadian system.

## Methods

### Rats

Male Sprague Dawley (SD) rats at 6–7 weeks of age were used for all experiments unless otherwise stated, maintained at 20–24 °C with unrestricted access to rodent chow and water. They were housed under a 12 h light:12 h dark (LD) cycle (lights on 07:00–19:00), or in constant darkness for the last 2 days (DD). Animals were sacrificed by rising CO_2_ and cervical dislocation unless otherwise stated. For polymerase chain reaction (PCR), quantitative PCR (qPCR) and western blotting, pineal glands were dissected and rapidly frozen on dry ice. For PCR analysis, adult male SD rats were sacrificed at zeitgeber time (ZT) 2, where ZT0 corresponds to 07:00. For western blotting analysis, adult male or female SD rats were sacrificed at ZT2 or 7. For immunohistochemistry, rats were sacrificed by intraperitoneal (IP) injection of pentobarbital at ZT5 and transcardially perfused with 4% paraformaldehyde in phosphate buffer, the pineal glands removed and immersed in the same fixative overnight at 4 °C. To determine whether RA signaling genes exhibit diurnal changes in expression, pineal glands were collected at 6 h intervals over 24 h (h), ZT0, 6, 12 and 18, and gene expression determined by qPCR. At ZT12 and 18, which occurred during the dark period, dissections were performed under dim red light. To determine whether changes in RA signaling genes are driven by the light/dark cycle, rats were housed in constant darkness for 2 days and pineal glands were collected under dim red light at 6 h intervals over 24 h (circadian times (CT) 0, 6, 12 and 18; CT0 and CT12 correspond to subjective light on and light off, respectively). All animal procedures were carried out in accordance with Home Office regulations and local ethics committee guidelines.

### PCR

Total RNA was extracted from individual pineal glands using an RNeasy mini kit (Qiagen) with on-column DNase digestion (Qiagen). RNA was quantified using a NanoDrop spectrophotometer (Thermo Scientific) and precipitated in 100% ethanol, linear acrylamide and ammonium acetate. cDNA was synthesized using a High Capacity RNA-to-cDNA kit (Applied Biosystems). PCR analysis was performed using primers designed for *Rdh10*, *Rdh12*, *Raldh1*, *Raldh2*, *Raldh3*, *Rara*, *Rarb*, *Rarg*, *Stra6*, *Cyp26a1*, and *Cyp26b1* (Table 1a). PCR products were visualized by agarose gel electrophoresis and UV transillumination.

**Table 1:** Sequences of primers (a) and probes (b) used for rat RT-PCR and qPCR analyses. Probes were designed by PrimerDesign Ltd

a	Gene	RefSeq code	Product size (bp)	Forward primer (5′-3′)	Reverse primer (5′-3′)
	*Raldh1*	NM_022407.3	196	ACGTGGAAGAAGGGGACAAGGCTG	GCAAAGACTTTCCCACCATTGAGTGCC
	*Raldh2*	NM_053896.2	198	CAAGGAGGCTGGCTTTCCACCC	GGGCTCTTCCCTCCGAGTTCCA
	*Raldh3*	NM_153300.1	122	TGCCAAAACGGAGCAGGGGC	TGTCCTCCATGGCTGACCCCC
	*Rara*	NM_031528.2	215	CGCCAAGGGAGCTGAACGGG	GGGTGGCTGGGCTGCTTCTG
	*Rarb*	NM_031529.1	134	ACACCACGAATTCCAGCGCTGAC	CAGACCTGTGAAGCCCGGCA
	*Rarg*	NM_001135250.1	218	CCTGTGAAGGCTGCAAGGGCT	GTCTGGTGGGCCTTCTTCCT
	*Rdh10*	NM_181478.2	98	ACACGGGCATGTTCAGAGGCTG	GGCCTTCATGGCCTGCTTCACA
	*Rdh12*	NM_001108037.1	114	GAGCTGGCCAAGCGGCTCC	GAGGCGCCACAACAGGCACAT
	*Cyp26a1*	NM_130408.2	156	TTCGGGTGGCTCTGAAGACT	CCTCTGGATGAAGCGATGTAAAT
	*Cyp26b1*	NM_181087.2	127	TCCATTGGCGACATCCACCGC	GGCTGCTCCAGGCTCGAAGTG
	*Stra6*	NM_001029924.1	246	TTGTGCTTCGGCAGGGCACC	CTGGTCTGCAGCCCCTGGGA
	*Aanat*	NM_012818.2	122	GCGCGAAGCCTTTATCTCAGTCTCG	AGGCCACAAGACAGCCCTCCT
	*Bmal1*	NM_024362.2	135	CGGGCGACTGCACTCACACA	GCCAAAATAGCCGTCGCCCTCT
	*Gapdh*	NM_017008.4	119	GGGCTCTCTGCTCCTCCCTGT	CAGGCGTCCGATACGGCCAAA
b	Gene	RefSeq code	Product size (bp)	Sense	Anti-sense
	*Raldh1*	NM_022407.3	106	TTGAGAGTGGGAAGAAAGAAGGA	TCATCGGTCACATTGGAGAAGA
	*Raldh2*	NM_053896	80	GGGGGTTCAAGATGTCTGGAA	CGTCACGGTCTTTACTTCTGAG
	*Rara*	NM_031528	92	ATGCCACCACTTATCCAGGAA	CCACCCCCATCTCGTGTTC
	*Rarb*	NM_031529	115	AGTGCCATCTGCTTAATCTGT	GCCGTCGTTTTCTAATGTAAATC

### Western Blotting

Tissue samples were disassociated by mechanical homogenization in a homogenization buffer (150 mM NaCl, 1% Triton, 0.1% SDS, 50 nM HEPES) containing a broad-spectrum protease inhibitor cocktail which inhibits a broad spectrum of serine and cysteine proteases (Roche) and phosphatase inhibitor cocktail (Sigma). Protein concentration was measured by bicinchoninic acid (BCA) assay (Thermo Scientific). Protein samples were separated on 12% SDS-polyacrylamide gels (50 μg per lane for RALDH1, RALDH2, RARα, RARβ; 14 μg per lane for ERK1/2, phospho-ERK1/2; and 40 μg per lane for Akt and phospho-Akt). Following separation, proteins were transferred onto Hybond ECL nitrocellulose membrane (GE Healthcare). Membranes were blocked in 5% dried skimmed milk powder in tris-buffered saline containing 0.05% Tween-20 (TBST) for 1 h. Membranes were incubated overnight at 4 °C with antibodies against RALDH1, RALDH2, RARα, RARβ, ERK1/2, phospho-ERK1/2, Akt and phospho-Akt (Table 2). Antibodies were diluted in the blocking solution with the exceptions of phospho-Akt, which was diluted in 5% bovine serum albumin (BSA; Sigma-Aldrich) in TBST, and ERK1/2 and phospho-ERK1/2, which were diluted in 2% BSA in TBST. Following washes in TBST, membranes were incubated for 1 h at room temperature in species-appropriate horseradish peroxidase (HRP)-conjugated secondary antibodies diluted in blocking solution (1:5000; Jackson Immunoresearch). Membranes were washed again in TBST and protein bands visualized by enhanced chemiluminescence (Millipore) and exposure to X-ray film (RALDH1, RALDH2, RARα and RARβ) or imaged using a MyECL imager (ERK1/2, phospho-ERK1/2, Akt and phospho-Akt; Thermo Scientific). Band densities were quantified using ImageJ software.

**Table 2:** Primary antibodies used for western blot (WB) and immunohistochemistry (IHC). RALDH, retinaldehyde dehydrogenase; RAR, retinoic acid receptor; GFAP, glial fibrillary acidic protein

Antibody	Host	WB dilution	IHC dilution	Predicted molecular weight (kDa)	Source	Catalog #
Anti-RALDH1	Rabbit	1:1000	1:300	54	Abcam	ab24343
Anti-RALDH2	Rabbit	1:3000	–	55	Millipore	ABN420
Anti-RARα	Goat	1:1500	–	51	Abcam	ab28767
Anti-RARα	Rabbit	–	1:300	–	Santa Cruz	sc-551
Anti-RARβ	Rabbit	1:500	–	50	Abcam	ab53161
Anti-S-antigen	Mouse	–	1:50	–	Santa Cruz	sc-166383
Anti-GFAP	Mouse	–	1:300	–	Sigma-Aldrich	G3893
Anti-ERK1/2	Rabbit	1:2000	–	44/42	Cell Signaling	9102
Anti-phospho-ERK1/2	Rabbit	1:4000	–	44/42	Cell Signaling	4370
Anti-Akt1/2/3	Rabbit	1:1000	–	56/62	Santa Cruz	sc-8312
Anti-phospho-Akt	Rabbit	1:1000	–	60	Cell Signaling	9271

### Immunohistochemistry

Perfusion-fixed pineal glands were processed into paraffin wax blocks, sectioned at 7 μm onto polysine-coated slides and dried overnight at 37 °C. Sections were dewaxed in Histo-Clear (National Diagnostics) and rehydrated in decreasing ethanol concentrations. Antigen retrieval was performed by boiling the sections for 10 min in sodium citrate buffer, pH 6. Sections were labeled with antibodies against RARα, RALDH1, S-antigen (SAG) and glial fibrillary acidic protein (GFAP; Table 2), and appropriate fluorescent secondary antibodies. The slides were mounted using mounting medium containing bisbenzimide (Sigma-Aldrich) and labeling was visualized using fluorescence microscopy. Images were taken of randomly selected fields for quantification of labeled cells. The total number of RALDH1- and RARα-positive cells was assessed by counting labeled cells in a minimum of five images with at least 100 cells per image. The quantification of the number of cells double-labeled with RALDH1/RARα and SAG/GFAP was based on counts of 60–170 cells over a minimum of five images.

### Quantitative PCR

Total RNA was extracted from individual pineal glands using an RNeasy mini kit (Qiagen) with on-column DNase digestion (Qiagen). RNA was quantified using a NanoDrop spectrophotometer (Thermo Scientific) and precipitated in 100% ethanol, linear acrylamide and ammonium acetate. cDNA was synthesized from 500 ng total RNA using a High Capacity RNA-to-cDNA kit (Applied Biosystems). qPCR analysis was performed using SensiMix SYBR mastermix (Bioline) using primers designed for *Aanat*, *Bmal1*, *Rarg*, *Raldh3*, *Rdh10*, *Rdh12*, *Cyp26a1*, *Cyp26b1* and *Gapdh* (Table 1a) or using PrecisionPLUS MasterMix (PrimerDesign) and probes designed by PrimerDesign Ltd for *Raldh1*, *Raldh2*, *Rara*, *Rarb* and *Gapdh* (Table 1b). Samples were run on a LightCycler 480 (Roche) and data analyzed using LightCycler 480 Software 1.5. Target gene expression was normalized to *Gapdh* expression. The specificity of all primer sets towards the intended target was confirmed by sequencing of the PCR products by GATC Biotech, followed by checking the sequences against the rat RefSeq RNA database on NCBI BLAST.

### Pineal Gland Culture

Pineal glands were obtained for tissue culture at ZT 4–5 and were cultured based on the method used by Bailey et al. [35]. They were rapidly dissected and placed immediately into ice-cold culture medium consisting of BGJb medium (Fitton-Jackson modification; Gibco) containing 0.1% BSA, 25 mM HEPES buffer (Sigma-Aldrich), 2 mM GlutaMAX supplement (Gibco), 0.1 mg/ml ascorbic acid (Stem Cell Technologies) and 100 U/ml penicillin-streptomycin (Gibco). Meninges were removed under a dissection microscope and pineal glands were transferred onto Millicell culture plate inserts (Millipore) in a 6-well plate, one pineal gland per well. Pineal glands were incubated in 1 ml culture medium at 37 °C, 5% CO_2_; media was changed after 24 and 48 h. The pineal glands were treated after 48 h in culture. For gene expression experiments, treatments consisted of all-*trans*-RA (1 μM; Sigma-Aldrich), norepinephrine (NE; 1 μM; Sigma-Aldrich), or vehicle control for 4 h. For kinase activation experiments, treatments were all-*trans*-RA (1 μM), epidermal growth factor (EGF; 0.6 μg/ml; R & D Systems), or vehicle control for 10 min. RA and NE were dissolved in dimethyl sulfoxide (DMSO; Sigma-Aldrich), therefore control and EGF treatments received an equivalent concentration of DMSO (0.01%). Following treatment, pineal glands on the membrane inserts were rapidly frozen on dry ice and stored at − 70 °C until qPCR or western blotting analysis.

For the culture of pineal glands obtained from postnatal day (P) 10–12 rats, male SD rat pups were euthanized by IP injection of pentobarbital at ZT7 and the pineal glands were rapidly dissected and placed immediately into ice-cold culture medium consisting of 50% minimum essential medium (MEM; Sigma-Aldrich), 25% heat-inactivated horse serum (Sigma-Aldrich), 25% Hanks’ buffered salt solution (HBSS; Gibco), supplemented with 100 U/ml penicillin-streptomycin, 2 mM GlutaMAX supplement, 5 mg/ml additional glucose and buffered with 25 mM HEPES. Meninges were removed under a dissection microscope and pineal glands were transferred onto Millicell culture plate inserts in a 6-well plate, 2 to 3 pineal glands per well. The pineal glands were incubated in 1 ml culture medium at 35 °C, 5% CO_2_. After 24 h, the media was removed and replaced with serum-free vitamin A-deficient medium consisting of Neurobasal medium (Gibco), supplemented with vitamin A-deficient B-27 supplement (Gibco), 100 U/ml penicillin-streptomycin, 2 mM GlutaMAX supplement and 5 mg/ml additional glucose. Pineal glands were incubated for 3 days to allow for depletion of vitamin A and its metabolites. The medium was replaced with fresh vitamin A-deficient medium and pineal glands were treated with all-*trans*-RA (1 μM) or vehicle control for 4 or 48 h.

### Retinoic Acid Measurement

A RA reporter cell line (Sil-15) was used to measure RA production in rat pineal glands [44, 45], as the lipid nature of RA and nanomolar concentrations endogenously present in tissues make this compound difficult to detect by other means. The cell line is derived from F9 teratocarcinoma cells transfected with a transgene consisting of *LacZ* driven by multiple tandem copies of the RA response element (RARE) from the mouse *Rarb* promoter [46]. Sil-15 cells were grown in Dulbecco’s Modified Eagle Medium (Gibco) containing 10% fetal calf serum (Gibco) and 0.8 mg/ml G418 (Sigma-Aldrich), at 37 °C, 5% CO_2_. Cells were plated onto gelatin-coated 96-well plates and allowed to reach confluence before use. Pineal glands were collected from 6-week-old male SD rats at ZT0 and 12, sliced into three parts and transferred to 96-well plates (one pineal gland per well) in culture medium consisting of 50% MEM, 25% heat-inactivated horse serum, 25% HBSS, supplemented with 1 μM retinol (Sigma-Aldrich) to match circulating levels of retinol, 100 U/ml penicillin-streptomycin, 2 mM GlutaMAX supplement, 5 mg/ml additional glucose, and buffered with 25 mM HEPES. Pineal glands were incubated at 35 °C, 5% CO_2_ for 2 h, protected from light. The pineal gland-conditioned media and blank medium were diluted 1:3 in the culture medium before adding to the plated Sil-15 cells in triplicate under dim yellow light. A serial dilution of all-*trans*-RA was prepared and added to Sil-15 cells for generation of a standard curve. Sil-15 cells were incubated at 37 °C, 5% CO_2_ for 22 h. Cells were washed in PBS, fixed with 1% glutaraldehyde (Sigma-Aldrich) for 15 min, followed by additional PBS washes. LacZ expression was detected using X-gal solution (2 mg/ml 5-bromo-4-chloro-3-indolyl-β-D-galactopyranoside (X-gal; Promega), 3.3 mM potassium ferrocyanide, 3.3 mM potassium ferricyanide, 1 mM magnesium chloride) incubated at 37 °C, 5% CO_2_ for 24 h. The absorbance was measured on an Emax precision microplate reader (Molecular Devices). Mean absorbance readings were corrected for the mean blank medium absorbance and RA concentration interpolated from the cubic spline-fitted standard curve generated using GraphPad Prism version 5.04.

### Statistics

Data were analyzed by unpaired Student’s *t* test or one-way ANOVA with Tukey’s multiple comparison test as appropriate. In cases where data were not normally distributed, Mann-Whitney *U* or Kruskal-Wallis tests were performed as appropriate. Non-parametric data is plotted as median values with interquartile range.

## Results

### The Components Required for RA Signaling Are Present in the Rat Pineal Gland

**Fig. 1:**
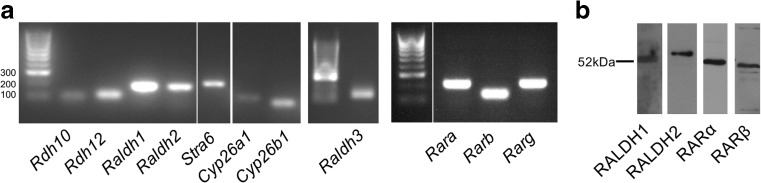
The components necessary for retinoic acid signaling are present in the rat pineal gland. mRNA expression of the genes encoding the synthetic enzymes retinol dehydrogenase, *Rdh10*, and the three retinaldehyde dehydrogenases, *Raldh1*, *Raldh2*, and *Raldh3*; the retinal reductase enzyme *Rdh12*; the retinol transporter *Stra6*; the retinoic acid receptors, *Rara*, *Rarb* and *Rarg*; and the catabolic enzymes cytochrome P450 family 26 (*Cyp26*) *a1* and *Cyp26b1*; determined by PCR and gel electrophoresis (**a**). Protein for RALDH1, RALDH2, RARα and RARβ is present, determined by western blotting (**b**)

For RA signaling to occur in a given tissue, the enzymes that synthesize RA from retinol and RARs must be present. An initial PCR screen was performed on the adult rat pineal gland to determine the expression of the genes encoding the required components of the RA signaling pathway. Transcripts encoding the retinol dehydrogenase (RDH) and retinaldehyde dehydrogenase (RALDH) enzymes that catalyze the first and second steps, respectively, of the conversion of retinol to RA were detected by PCR (Fig. 1a). Transcript encoding another member of the RDH family, *Rdh12*, was also detected, which primarily acts as a retinaldehyde reductase enzyme, converting retinaldehyde to retinol [47]. Genes encoding each of the three subtypes of RARs, *Rara*, *Rarb* and *Rarg*, were also found to be expressed. Transcripts encoding *Stra6*, the retinol transporter, and *Cyp26a1* and *Cyp26b1*, encoding the RA catabolic enzymes, were also detected. Western blotting confirmed the presence of RALDH1, RALDH2, RARα and RARβ protein in the adult rat pineal gland (Fig. 1b). This demonstrates that cells in the pineal gland have the potential to synthesize, respond to and degrade retinoic acid.

### Immunohistochemical Detection of RALDH1 and RARα in the Pineal Gland

**Fig. 2:**
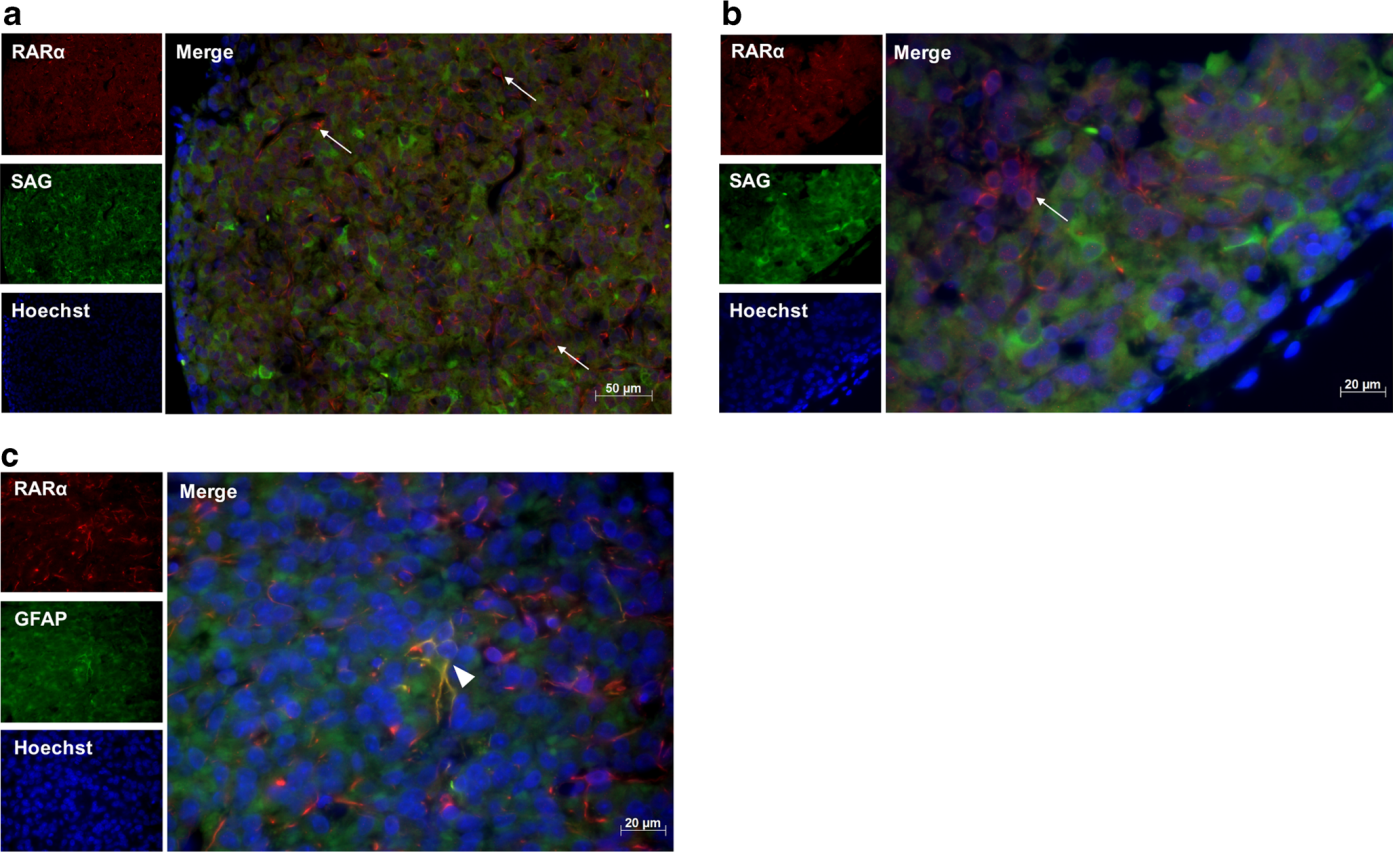
Immunohistochemistry for detection of RARα in the rat pineal gland. Representative images of double-labeling of paraffin sections of the rat pineal gland with antibodies against RARα and S-antigen (SAG; **a**, **b**) or GFAP (**c**), at × 20 (**a**) or × 40 (**b**, **c**) magnification. RARα-immunoreactivity was detected in the cytoplasm of cells throughout the pineal parenchyma, indicated by arrows. No co-localization was detected of RARα with SAG, whereas a small subset of RARα-positive cells co-expressed GFAP, indicated by arrowhead

**Fig. 3:**
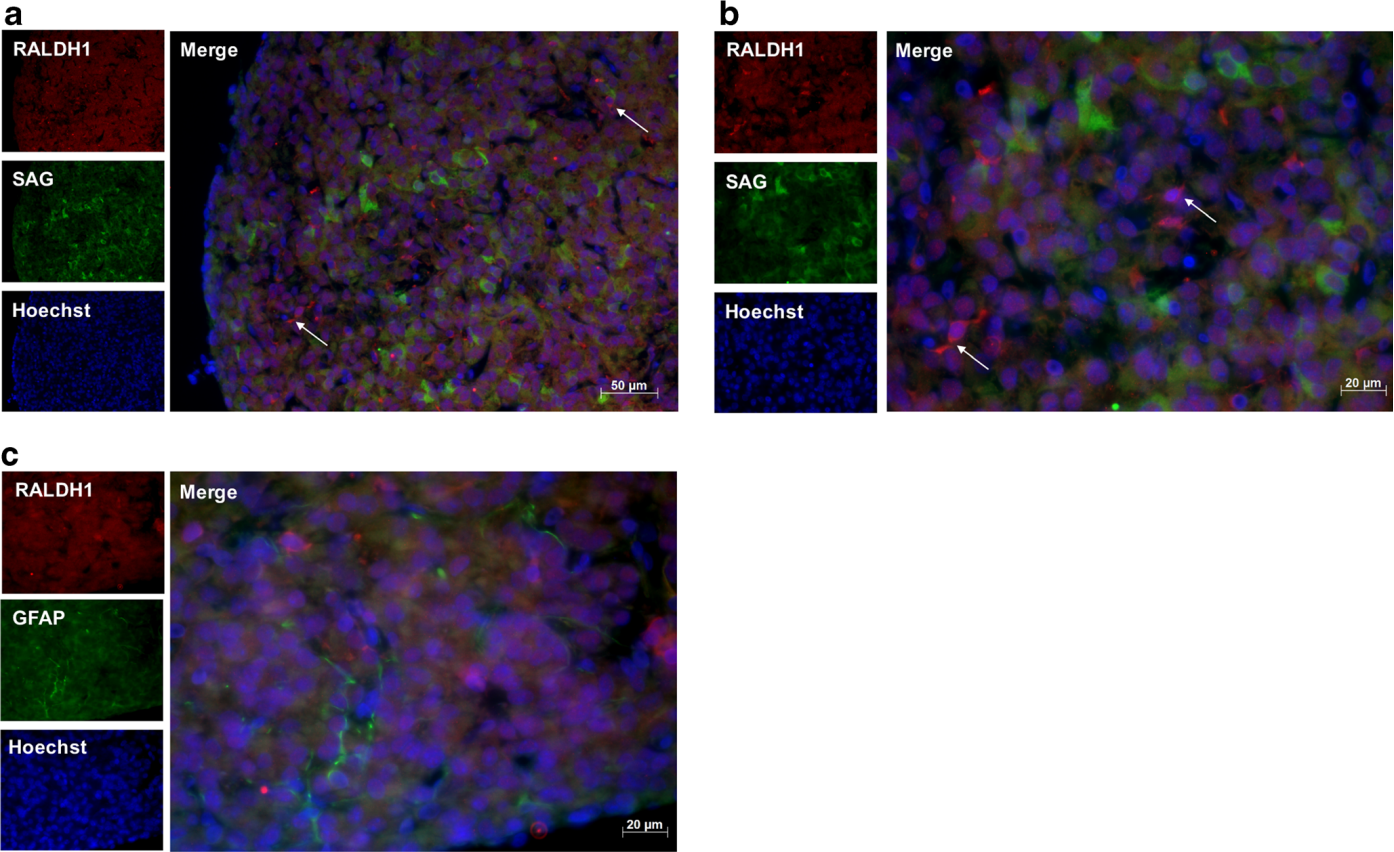
Immunohistochemistry for detection of RALDH1 in the rat pineal gland. Representative images of double-labeling of paraffin sections of the rat pineal gland with antibodies against RALDH1 and S-antigen (SAG; **a**, **b**) or GFAP (**c**), at × 20 (**a**) or × 40 (**b**, **c**) magnification. RALDH1-immunoreactivity was detected in the cytoplasm of cells throughout the pineal parenchyma, indicated by arrows. No co-localization was detected of RALDH1 with SAG or GFAP

The components required for RA synthesis and signaling were found to be present in the pineal gland, therefore their cellular localization was determined for those of which the antibodies worked by immunohistochemistry; this was the case for antibodies recognizing the enzyme RALDH1 and the receptor RARα. The pinealocyte is the principal cell type in the pineal gland, comprising more than 95% of cells [48]. Interstitial cells resembling astrocytes are also present, several of which express GFAP [49]. To determine which cell type express RARα and RALDH1, pineal gland sections were double-labeled with antibodies against SAG, a pinealocyte marker [48], and GFAP. RARα-immunoreactivity was detected in the cytoplasm of cells throughout the pineal parenchyma, with an average of 14% of cells labeled (Fig. 2). None of these cells were found to co-express SAG, however a small subset of 1% of RARα-positive cells co-expressed GFAP (Fig. 2c). RALDH1-immunoreactivity was also detected in the cytoplasm of cells, with a uniform distribution throughout the pineal gland sections and an average of 6% of cells labeled (Fig. 3). There was no double-labeling of RALDH1 with SAG or GFAP detected. These results suggest that RARα and RALDH1 are predominantly present in the cytoplasm of SAG-negative pinealocytes, given the number of immunoreactive cells present and the lack of co-expression with GFAP, with a small proportion of RARα also present in GFAP-positive cells.

### Diurnal Changes in the Expression of RA Signaling Genes

**Fig. 4:**
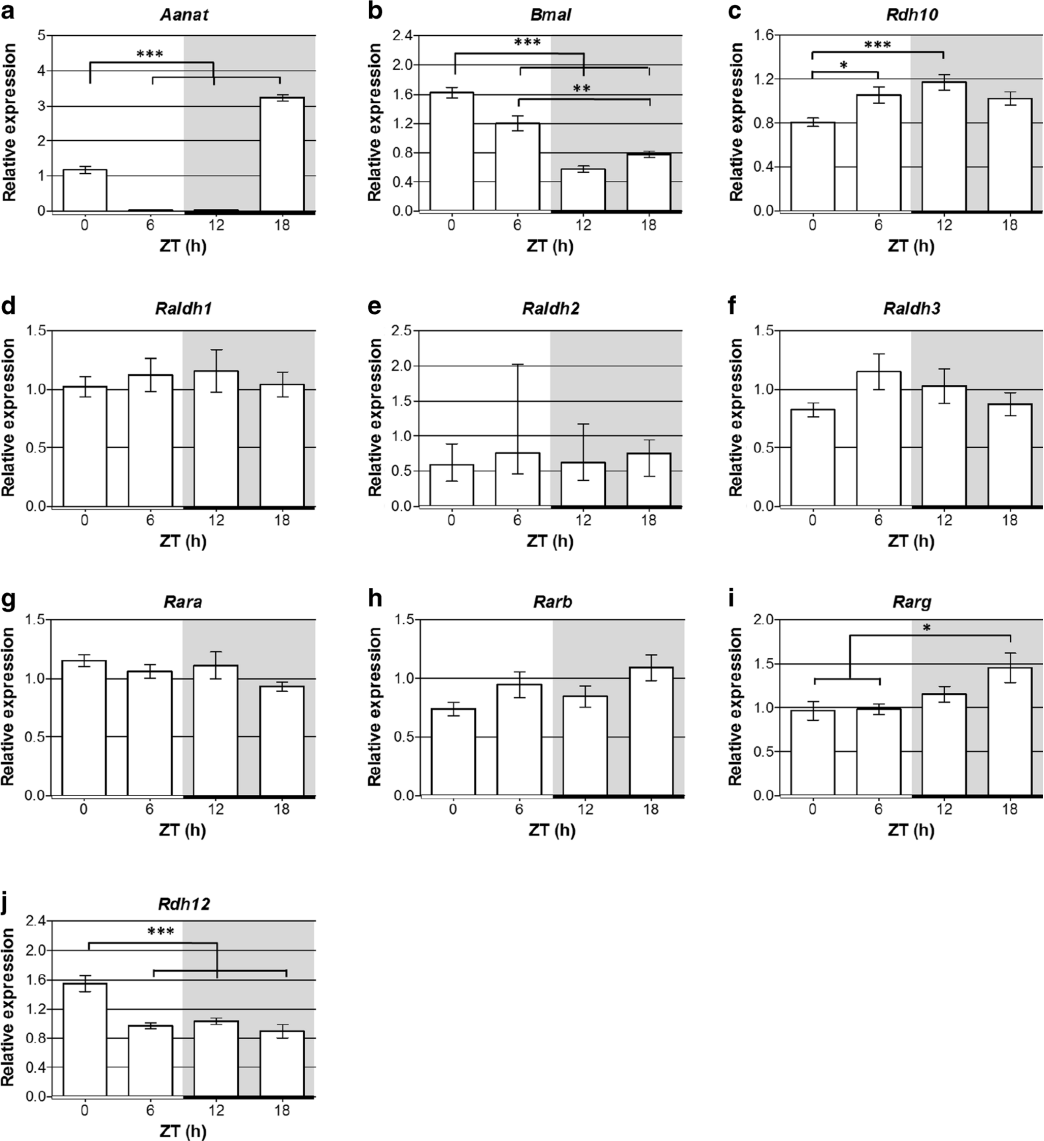
Retinoic acid signaling genes exhibit diurnal changes in expression in the rat pineal gland. qPCR analysis of expression of *Aanat*, *Bmal1* and retinoic acid signaling genes in Sprague Dawley rat pineal glands collected at different zeitgeber times (ZT) throughout a 24 h cycle; the horizontal bar represents the light/dark cycle, white bar = light phase, black bar = dark phase; grey shading indicates the dark period. Values represent mean mRNA expression relative to *Gapdh*, ± SEM; with the exception of *Raldh2* which represent median mRNA expression relative to *Gapdh,* with interquartile range, as these data are not normally distributed. *N* ≥ 6 glands per time-point. *P < 0.05; **P < 0.01; ***P < 0.001

The expression of RA signaling genes over the 24 h light/dark cycle were investigated to determine if RA signaling is subject to diurnal changes. Expression of the genes encoding the RA receptors (*Rara*, *Rarb* and *Rarg*) and the enzymes for the first (*Rdh10* and *Rdh12*) and second (*Raldh1, Raldh2*, and *Raldh3*) steps of RA synthesis were measured by qPCR at ZT0, 6, 12 and 18. *Aanat*, the gene encoding the melatonin synthetic enzyme AANAT, was used as a positive control and its mRNA expression showed a peak at the expected time of ZT18 (Fig. 4a) [50]*.* A circadian clock gene was also examined as a second positive control, *Bmal1*, which peaked at ZT0–6 (Fig. 4b), as previously reported for the rat pineal gland [51].

The genes required for RA signaling all showed some level of expression throughout the light/dark cycle (Fig. 4c–j) suggesting that a background level of RA signaling is present at all times. Several of these genes showed significant diurnal changes in expression. The expression of *Rdh10* was lowest at the start of the light period at ZT0 and peaked at ZT12, the start of the dark period (Fig. 4c); this gene encodes the enzyme required for the first step of the conversion of retinol to RA. At the following time point sampled, ZT18 the mid-point of the dark period, the *Rarg* gene peaked, increasing in expression following its lowest expression during the day at ZT0–6 (Fig. 4i). This gene encodes one of the RA receptors necessary to transduce the RA signal. Another member of the RDH family, *Rdh12*, which reduces and so removes retinaldehyde available for RA synthesis [47], peaked at ZT0 at the end of the dark period (Fig. 4j), then decreased at ZT6 and remained low at ZT12 and ZT18. These results show there are diurnal changes in the expression of RA signaling genes at each stage of the signaling pathway that may collectively complement each other to promote diurnal changes in RA production and activity.

### Diurnal Change in RA Production

**Fig. 5:**
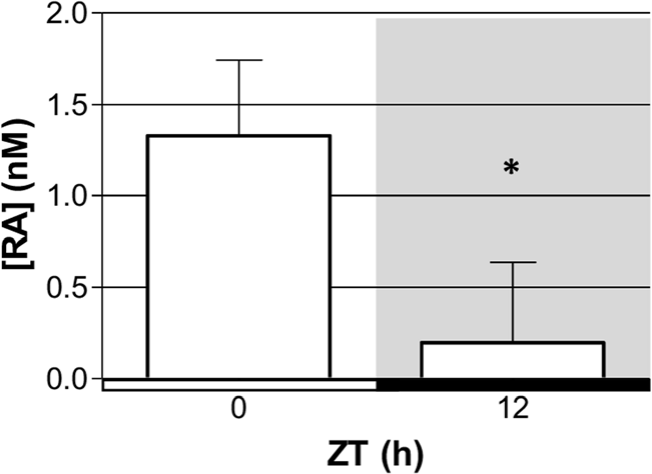
Production of retinoic acid in the rat pineal gland exhibits a diurnal change. Retinoic acid concentration [RA] measured in spent media from pineal glands collected at zeitgeber time (ZT) 0 and 12 and cultured for 2 h, measured by a retinoic acid reporter cell line. The horizontal bar represents the light/dark cycle, white bar = light phase, black bar = dark phase; grey shading indicates the dark period. Values represent median with interquartile range. *N* = 4 glands per time-point. *P < 0.05

The components required for RA production are present in the rat pineal gland at the mRNA and protein level. RA was measured at two time-points to confirm that the pineal gland synthesizes RA and to determine whether RA production is subject to diurnal changes. RA release was measured from pineal glands collected at the start and end of the light period, ZT0 and ZT12, respectively. Pineal glands were cultured for 2 h immediately following dissection to allow release of RA into the medium, followed by analysis of RA concentration in the spent media by a RA reporter cell line. RA was detected at both time-points and was found to be significantly higher at ZT0 compared to ZT12, with a sevenfold difference in concentration (Fig. 5). These results demonstrate that the pineal gland is capable of producing RA and this is subject to diurnal changes, with higher levels present at the start of the light period compared to the end.

### Diurnal Changes in RA Activity

**Fig. 6:**
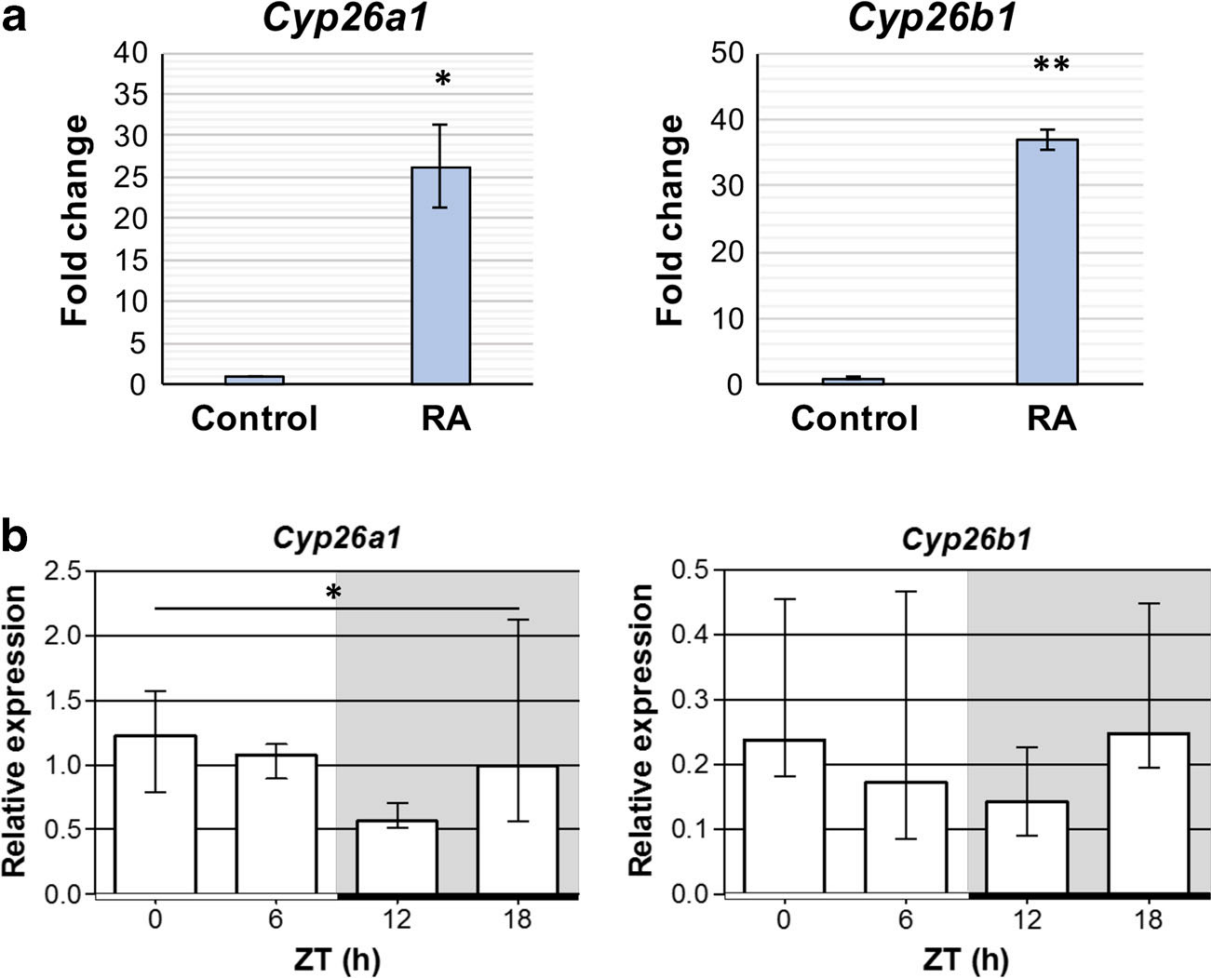
Diurnal changes in *Cyp26a1* expression suggest there are diurnal changes in retinoic acid activity in the rat pineal gland. **a** Retinoic acid (RA) rapidly induces upregulation of RA-responsive genes *Cyp26a1* and *Cyp26b1* in the rat pineal gland. qPCR analysis of cultured rat pineal glands following 4-h treatment with vehicle control or RA. Values represent the fold change in mean mRNA expression compared to control, ± SEM. *N* = 3 glands per treatment. *P < 0.05; **P < 0.01. **b** *Cyp26a1* exhibits diurnal changes in expression in the rat pineal gland. qPCR analysis of Sprague Dawley rat pineal glands collected at different zeitgeber times (ZT) during the light/dark cycle; white bar = light phase, black bar = dark phase; grey shading indicates the dark period. Values represent median mRNA expression relative to *Gapdh*, with interquartile range. *N* ≥ 6 glands per time-point. *P < 0.05 (Kruskal-Wallis test)

Significant diurnal changes in the expression of several genes encoding RA signaling proteins and in RA production suggest that RA signaling is subject to diurnal changes. RA is a potent regulator of gene transcription, therefore to investigate changes in RA activity, diurnal changes in two target genes, *Cyp26a1* and *Cyp26b1*, were determined. These genes encode the RA catabolic enzymes and have been shown to be potently induced by RA [52]. Firstly, *Cyp26a1* and *Cyp26b1* were shown to be rapidly upregulated by RA in cultured ex vivo rat pineal glands (Fig. 6a). A 4-h RA treatment induced a 26- and 37-fold increase in expression of *Cyp26a1* and *Cyp26b1*, respectively. This demonstrates that these genes are responsive to RA in the rat pineal gland and are therefore indicators of RA activity. qPCR analysis of diurnal changes in expression of these genes found that overall there are significant differences in *Cyp26a1* (*P* = 0.0446; Fig. 6b), and it appears that the lowest expression is at ZT12, the start of the dark period. *Cyp26b1* expression exhibited a similar trend though this was not statistically significant (*P* = 0.2809). This suggests a diurnal change in RA activity occurs endogenously in the rat pineal gland.

### Changes in Expression in Constant Darkness

**Fig. 7:**
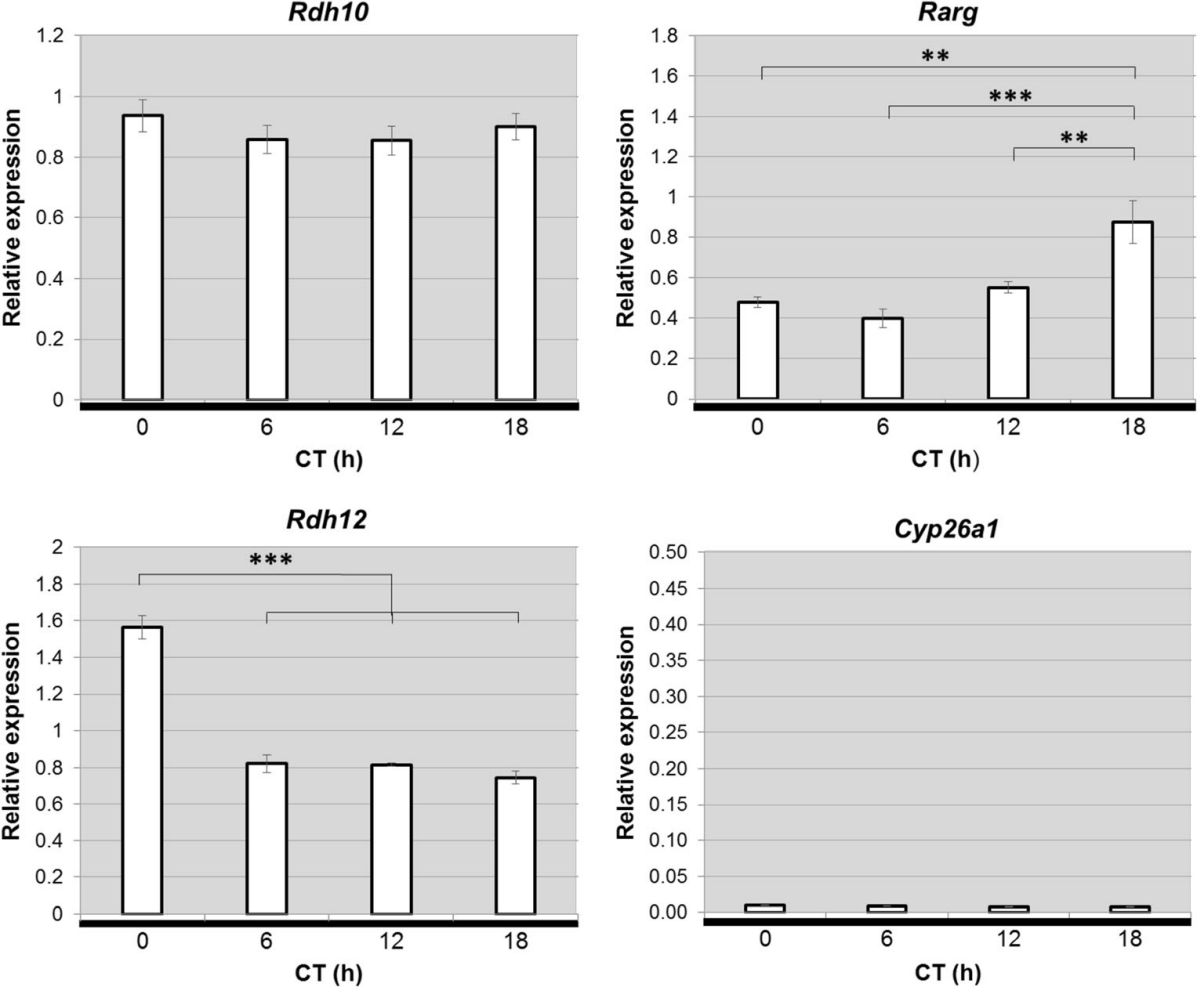
Diurnal changes in retinoic acid signaling genes, *Rarg* and *Rdh12*, persist in constant darkness in the rat pineal gland. qPCR analysis of expression of retinoic acid signaling genes in pineal glands of Sprague Dawley rats maintained in constant darkness for 2 days. Pineal glands were collected at different circadian times (CT) throughout a 24 h cycle; CT0 and CT12 correspond to subjective light on and light off, respectively; horizontal black bar and grey shading indicate the dark period. Values represent mean mRNA expression relative to *Gapdh*, ± SEM. *N* = 5 glands per time-point. **P < 0.01; ***P < 0.001

The RA signaling genes *Rdh10*, *Rarg*, *Rdh12* and *Cyp26a1* were shown to be differentially expressed between day and night. To determine if these changes persist in the absence of external light cues, expression of these genes over 24 h in the pineal glands of rats maintained in constant darkness were analyzed (Fig. 7). In constant darkness, *Rarg* and *Rdh12* maintained the same patterns of expression as in a normal LD cycle. In contrast, *Rdh10* expression, which displayed significant variation under LD conditions (Fig. 4), did not vary under conditions of constant darkness. *Cyp26a1* expression was very low at all time-points sampled, suggesting that expression is reduced under constant darkness compared to LD. The finding that the diurnal changes in *Rarg* and *Rdh12* persist in constant darkness suggest that they are driven by the endogenous circadian pacemaker, whereas the differences observed in the patterns of *Rdh10* and *Cyp26a1* expression between the LD and DD conditions suggest that the diurnal changes in these genes require input from the external light/dark cycle.

### NE Regulation of RA Signaling Genes

**Fig. 8:**
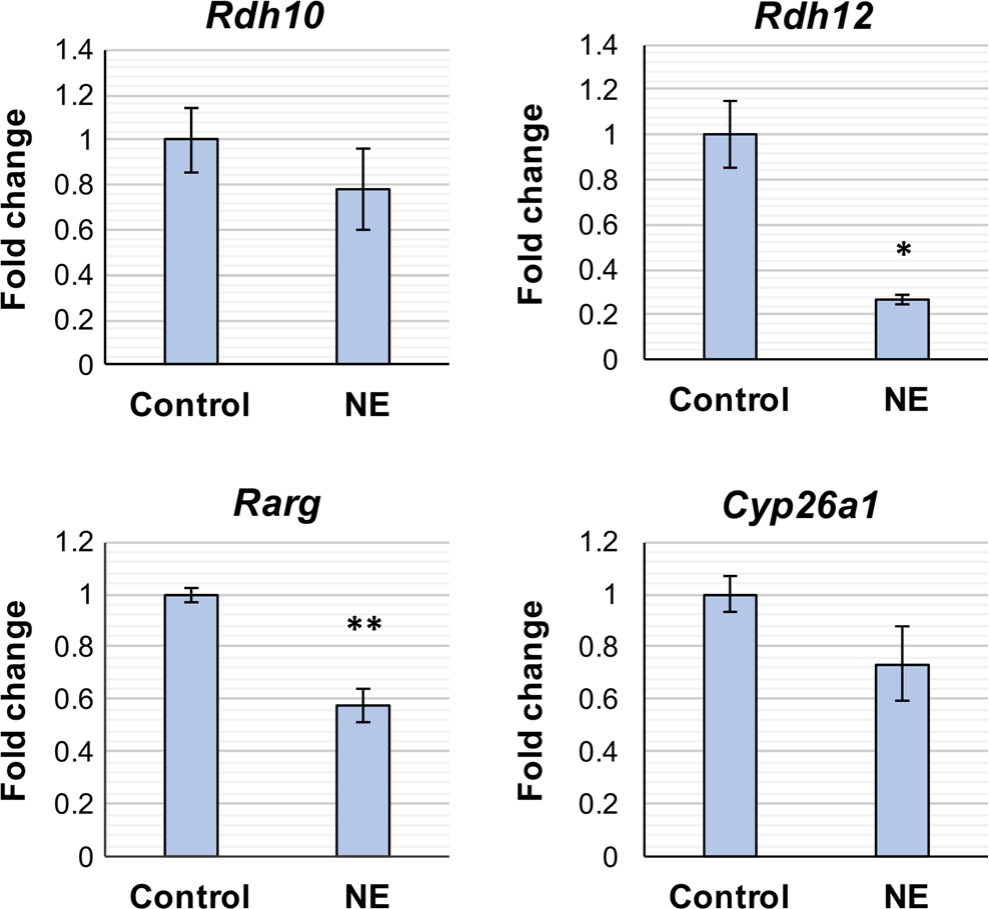
Norepinephrine represses *Rarg* and *Rdh12* gene expression. qPCR analysis of cultured rat pineal glands following 4-h treatment with vehicle control or norepinephrine (NE). Values represent the fold change in mean mRNA expression compared to control, ± SEM. *N* ≥ 3 glands per treatment. *P < 0.05; **P < 0.01

The majority of genes that cycle between day and night in the rat pineal gland are regulated by NE [35], therefore it was investigated whether the RA signaling genes that showed diurnal changes in expression in vivo*,* (*Rdh10*, *Rdh12*, *Rarg*, and *Cyp26a1*) are regulated by NE in vitro. Cultured pineal glands were treated with 1 μM NE for 4 h and gene expression determined by qPCR (Fig. 8). *Rdh12* and *Rarg* were responsive to NE and demonstrated a decrease in expression of 3.7- and 1.7-fold, respectively. There were no changes in *Rdh10* or *Cyp26a1* expression in response to NE (*P* = 0.3906 and 0.1692, respectively).

### Effect of RA on *Aanat* Induction

**Fig. 9:**
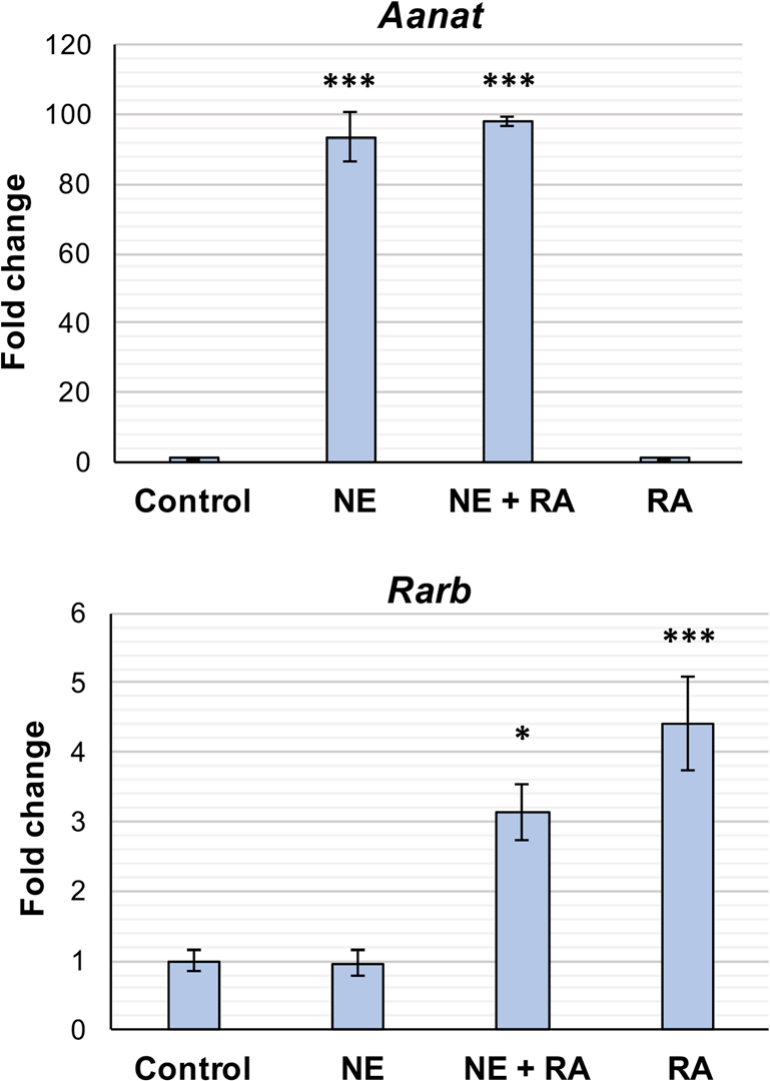
Retinoic acid does not rapidly influence *Aanat* transcription. qPCR analysis of cultured rat pineal glands following 4-h treatment with vehicle control, norepinephrine (NE), NE + retinoic acid (RA), or RA. Values represent the fold change in mean mRNA expression compared to control, ± SEM. *N* = 3–4 glands per treatment. *P < 0.05; ***P < 0.001, compared to control treatment

VAD has previously been shown to reduce the peaks of the rhythms in AANAT activity and melatonin in the rat pineal gland [26, 27]. In rodents, the AANAT rhythm is predominantly under transcriptional control with an increase in *Aanat* mRNA of more than a 150-fold at night in rats [25]. This study has demonstrated that RA, the active metabolite of vitamin A, is produced by the pineal gland on a diurnal basis and the components necessary for RA signaling are present, therefore the effect of RA on *Aanat* transcription was determined. Cultured pineal glands were treated with vehicle control, 1 μM RA or 1 μM NE for 4 h and gene expression analyzed by qPCR. As expected, *Aanat* responded robustly to treatment with NE, with expression increasing by 93-fold (Fig. 9), but there was no effect of RA on *Aanat*. As NE is the endogenous mediator of *Aanat* induction, the effect of RA combined with NE was also determined. Treatment of pineal glands with NE and RA induced an increase in *Aanat* comparable to treatment with NE alone, suggesting that RA does not influence NE-mediated induction of *Aanat*. Expression of the RA-responsive gene, *Rarb*, was also determined as a positive control. This increased in response to treatments of both RA and NE combined with RA, with comparable expression observed between these two treatments, indicating that RA is active. Taken together, these results suggest that RA does not have a short-term effect on *Aanat* transcription in the pineal gland.

**Fig. 10:**
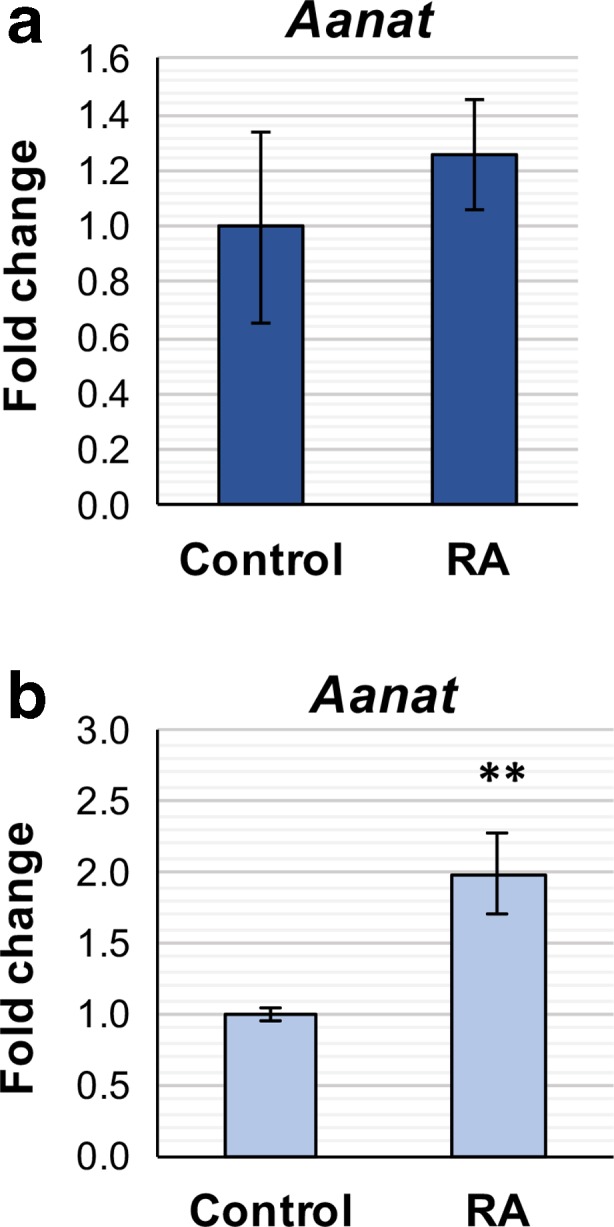
Long-term retinoic acid treatment induces upregulation of *Aanat* transcription. qPCR analysis of cultured P10–12 rat pineal glands following 4-h (**a**) or 48-h treatment (**b**) with vehicle control or retinoic acid (RA). Values represent the fold change in mean mRNA expression compared to control, ± SEM. *N* = 3 (**a**) or 6 (**b**) glands per treatment. **P < 0.01

RA can also regulate gene expression indirectly, through upregulation of the expression of transcription factors [53]. RA may act through such a mechanism to influence *Aanat* transcription in the pineal gland, however this would require a longer treatment period for an effect to be observed. This was investigated using pineal glands obtained from P10–12 rats. Firstly, it was confirmed that a short-term RA treatment does not influence *Aanat* induction in this culture model, as was previously observed in cultured pineal glands from older rats. Cultured pineal glands were treated with vehicle control or 1 μM RA for 4 h and gene expression analyzed by qPCR. As observed previously, there was no effect of RA on *Aanat* transcription following the 4-h treatment period (Fig. 10a). The effect of long-term RA treatment on *Aanat* was then tested by treating pineal glands with 1 μM RA for 48 h followed by qPCR analysis. In response to RA, *Aanat* was significantly increased by twofold relative to vehicle control (Fig. 10b). These results suggest that long-term RA treatment can influence *Aanat* transcription.

### RA Regulation of Kinase Activation

**Fig. 11:**
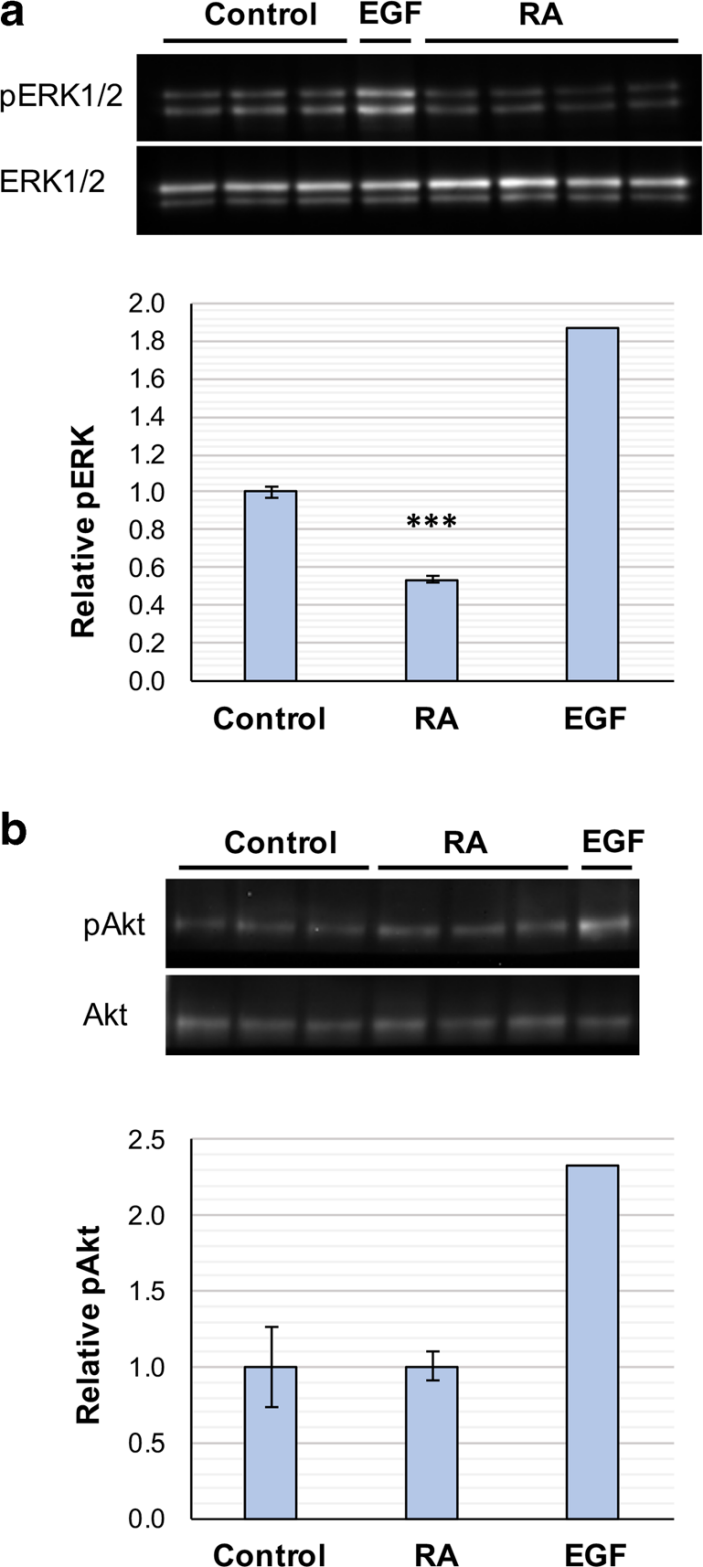
Retinoic acid rapidly downregulates ERK phosphorylation. Activation of ERK1/2 (**a**) and Akt (**b**) was determined by western blotting following 10 min treatment of cultured rat pineal glands with vehicle control or retinoic acid (RA). EGF was tested as a positive control (*n* = 1). Phosphorylated levels were normalized to total ERK or Akt; values represent the fold change in mean compared to control, ± SEM. *N* ≥ 3 glands per treatment. ***P < 0.001, compared to control

In the pineal gland, there is a daily rhythm in ERK1/2 phosphorylation with a peak during the night [26, 38] and vitamin A is required for this rhythm [26]. RA can modulate ERK1/2 phosphorylation in a number of cell lines and primary neuronal cultures [6, 54–56] and has been shown here to exhibit diurnal changes in signaling in the pineal gland, suggesting it may have a role in driving the daily rhythm in ERK1/2 activation. The effect of RA on kinase phosphorylation in the pineal gland was therefore determined. Cultured pineal glands were treated for 10 min with 1 μM RA, vehicle control or EGF as a positive control, and phosphorylated and total protein levels of ERK1/2 and Akt determined by western blotting. As expected, EGF induced activation of both ERK1/2 and Akt as previously reported [57]. RA treatment resulted in a significant decrease in ERK1/2 phosphorylation of nearly 50% (Fig. 11a), with both ERK1 and ERK2 individually exhibiting a comparable change (data not shown). There was no change in Akt phosphorylation in response to RA (Fig. 11b). These results demonstrate that RA can rapidly downregulate ERK1/2 phosphorylation in the rat pineal gland.

## Discussion

Previous studies have suggested an important role for vitamin A in circadian rhythms; its deficiency leads to disruption of daily rhythms in locomotor activity and clock gene expression [14–16], as well as reduction of the nocturnal peak in melatonin in the pineal gland and loss of rhythm in MAPK activation [26, 27]. The results presented here show that RA, the potent active metabolite of vitamin A, is subject to diurnal changes in production and activity, and it can induce rapid changes in kinase activation in the rat pineal gland.

**Fig. 12:**
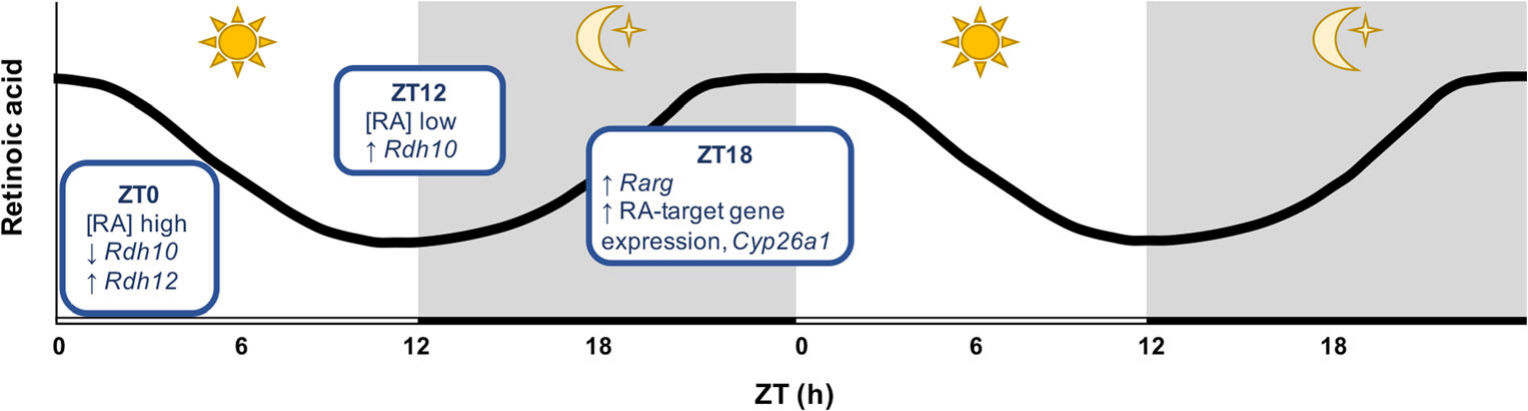
Summary schematic of the proposed retinoic acid rhythm in the rat pineal gland. At the start of the light period, zeitgeber time (ZT) 0, measured retinoic acid (RA) concentration is at its highest. At this time, the expression of the rate-limiting enzyme for the conversion of retinol to retinoic acid, *Rdh10*, is lowest. While there is a peak in the expression of *Rdh12*, which encodes an enzyme that converts retinaldehyde to retinol, therefore reducing the amount of retinaldehyde available for conversion to RA. These changes in expression of the RA synthetic enzymes are likely to lead to the sevenfold reduction in RA synthesis during the day, resulting in the low RA concentration measured at ZT12. At this time, *Rdh10* expression rises to its peak, which is expected to increase RA synthesis again during the night, returning RA concentration to the high levels detected at the end of night, at ZT0. This may be accompanied by an increase in RA signaling during the night, with a peak in expression of *Rarg* at ZT18, which encodes one of the RA receptors, and increase in expression of the RA-responsive gene, *Cyp26a1*

There are several lines of evidence presented here for rhythmic diurnal RA synthesis and signaling in the pineal gland (summarized in Fig. 12). Analysis of diurnal changes in expression of the RA signaling genes demonstrated significant changes at every stage of the signaling pathway, including RA synthesis from retinol, signal transduction and RA degradation. Furthermore different components of the pathway exhibit complementary patterns of gene expression which appear to act together to produce significant changes in RA activity. *Rdh10*, which encodes the rate-limiting enzyme required for RA synthesis from retinol [58], was found to be lowest at ZT0 and to peak at ZT12, the start of the dark period. This would be expected to lead to an increase in RA synthesis from retinol. At the next time point sampled, ZT18 the midpoint of the dark period, the gene encoding one of the RARs, *Rarg*, was found to peak, which may act to increase RA signaling to coincide with a rise in RA. At the end of the dark period, ZT0, there was a peak in *Rdh12* expression, this encodes a retinal reductase enzyme that converts retinaldehyde to retinol [47], therefore reducing the amount of retinaldehyde available for conversion to RA, which may act to bring down RA levels at the end of the night. This coincided with a decrease in both *Rdh10* and *Rarg* expression at ZT0. Significant diurnal changes were also observed in *Cyp26a1* expression, a RA-responsive gene and therefore an indicator of RA activity, which suggest an increase in RA signaling during the night. This gene encodes one of the RA catabolic enzymes and diurnal changes in its expression are also likely to lead to changes in RA degradation, assuming they are translated to changes at the protein level.

The combined effect of the changes in different components of the RA signaling pathway at complementary time-points appear to have a significant effect on RA synthesis, despite being relatively small changes in gene expression; measurement of RA production detected a sevenfold higher concentration at the end of the night compared to the start. This could be due to multiple components of the same signaling system acting in a concerted way leading to an additive effect. It is also possible that other compounds required for RA synthesis that were not measured here are changing diurnally to contribute to the diurnal change in synthesis. Although there is a transient postprandial peak in serum retinol, limited evidence suggests that there is no diurnal variation in circulating levels [59, 60], however diurnal changes in retinoid binding proteins have been reported. Plasma levels of the carrier for retinol in the circulation, retinol binding protein 4 (RBP4), have been shown to oscillate across the light/dark cycle in mice [61]. Furthermore, expression of the gene encoding the retinol transport protein, cellular retinol binding protein 1 (CRBP1), is under circadian control and peaks during the subjective night in liver [62]. Also expression of *Ttr*, which encodes another retinol carrier protein through its association with RBP, has been shown to increase significantly during the night in the rat pineal gland [35], which may act to increase retinol uptake during the night.

This is the first study to investigate diurnal changes in RA synthesis in the pineal gland or any other tissue. Bailey et al. [35] previously identified T3/RA signaling as one of four specialized functional gene groups in the rat pineal gland following a large-scale transcriptomic analysis, highlighting the importance of RA synthesis in this gland. However, the study did not pick up the relatively small changes in expression of the RA signaling genes that were observed here, most likely because they were below the threshold used to keep the false discovery rate low. *Rdh12* was highlighted however, for being highly expressed in the pineal gland relative to other tissues. Similarly, changes were seen in *Rorb*, which encodes a nuclear receptor that binds RA with high affinity [20]. This was found to increase in expression during the night at ZT18, in line with previous studies [22, 23]. This could act to increase RA activity during the night, consistent with the finding presented here of a peak in *Rarg* expression also at ZT18. An increase in the expression of retinoid receptors during the night has also been reported in the rat hippocampus; *Rara*, *Rarb* and *Rxrb* display circadian rhythms in expression which may be driven by direct transcriptional control by clock proteins via clock-responsive E-box sites in their regulatory regions [15].

The day/night variations in *Rdh12* and *Rarg* expression persisted under constant darkness, indicating that they are circadian in nature and driven by the endogenous circadian clock. This finding was supported by the result that NE regulates their expression in vitro. The nightly release of NE to the pineal gland from neuronal input from the superior cervical ganglia (SCG) is controlled by indirect innervation from the SCN, the site of the master clock, and is the primary regulator of circadian changes in gene expression in the pineal gland [35, 63]. Therefore, it is likely that the circadian changes in *Rdh12* and *Rarg* are driven by this input. In contrast, the diurnal changes in *Rdh10* and *Cyp26a1* were abolished under constant darkness at the time-points sampled, and these genes were found to be unresponsive to NE in vitro. This suggests that an alternative mechanism regulates the diurnal changes in *Rdh10* and *Cyp26a1*, independent of the SCN-driven NE input, but which requires input from the external light/dark cycle. In the case of *Cyp26a1* this is presumably RA itself and that RA synthesis is reduced under constant darkness and no longer cycles diurnally. Guillaumond et al. [26] reported that vitamin A is necessary for a diurnal rhythm in MAPK activation in the rat pineal gland by a mechanism that is independent of the SCG but that does require an intact SCN, demonstrating that an NE-independent route is important for the effects of vitamin A on pineal gland rhythms. This may also be important for generating the rhythm in RA synthesis. These findings suggest that the rhythmic RA signaling in the pineal gland is under the control of at least two modes of regulation—one that is driven by the circadian clock and another that is reliant on input from the external light/dark cycle. The RA rhythm may therefore constitute a unique rhythmic signaling system in the pineal gland, as other rhythmic events are almost exclusively driven by the rhythmic release of NE [35].

The diurnal rhythm in RA synthesis observed here makes it well-suited for a role in the rhythmic functioning of the pineal gland. Activation of ERK1/2 is subject to diurnal oscillations in the rat pineal gland, with an increase in levels of the phosphorylated forms of ERK1/2 shortly following onset of darkness [26, 38]. Vitamin A is required for this molecular rhythm [26], but it is currently unknown how its effects are mediated. RA was shown here to downregulate ERK1/2 phosphorylation in cultured pineal glands, therefore it may be involved in driving the rhythmic activation in vivo. There is a decrease in ERK1/2 activation towards the end of the night [26] when RA levels were found to be high, while lower RA levels at the start of night may permit the upregulation in ERK1/2 phosphorylation. ERK1/2 is involved in the regulation of the circadian clock in the pineal gland [39, 40] and there is also evidence that it serves a modulatory role in the nightly increase in AANAT activity [64], therefore its rhythmic activation may be an important signaling event for the generation of the rhythm in melatonin production.

The rapid effect of RA on ERK1/2 phosphorylation observed here implies a non-genomic activity of RA. Previous studies have shown that such effects of RA are mediated by extranuclear RARs, located in the cytoplasm and plasma membrane [6, 65, 66]. Immunohistochemical staining for RARα demonstrated strong expression localized to the cytoplasm, supporting a non-genomic role for RA signaling through RARα. Although no co-localization was observed with the pinealocyte marker SAG, it is likely that both the RARα-immunoreactive and RALDH1-immunoreactive cells are pinealocytes, given their morphology and number; over 95% of cells in the pineal gland are pinealocytes [67]. Pinealocytes are a heterogeneous cell population, and SAG is only present in a subset despite it being an established pinealocyte marker [48]. Therefore, RA appears to be produced and signal through RARα in the principal cell type of the pineal gland, the melatonin-synthesizing pinealocyte.

In the present study, induction of *Aanat* transcription was not responsive to short-term treatment with RA for 4 h, when administered both alone and in combination with NE. This suggests that the previously published effects of vitamin A on AANAT activity are not mediated by a rapid transcriptional effect of RA on *Aanat*. However, RA was found to induce upregulation of *Aanat* following a long-term treatment of 48 h. This suggests that RA is acting through an indirect mechanism to influence *Aanat* expression, such as through upregulation of the expression of intermediate transcription factors, which would require a longer treatment period. Indeed, RA induces upregulation of *cone-rod homeobox* (*Crx*) expression in retinoblastoma cells [68], a pineal- and retina-specific transcription factor which is required for the intact diurnal rhythm in *Aanat* mRNA [69]. RA has also been shown to stimulate acetylserotonin O-methyltransferase (ASMT) mRNA and enzyme activity, the enzyme that catalyzes the final step of melatonin synthesis [70], indicating that RA may have a long-term influence to increase melatonin synthesis through modulation of this enzyme and AANAT.

It is worth noting that the established method for pineal gland culture that was employed here and that has been widely and historically used to study pineal gland function in vitro uses a vitamin A-deficient medium [71]. It is likely that the absence of vitamin A would have a significant effect on signaling, given the present findings and that RA is a potent regulator of transcription in addition to its non-genomic effects. Indeed, Bailey et al. [35] reported differences in gene expression following culture which may be due to the absence of vitamin A. The results presented here demonstrate that RA is transcriptionally active in the pineal gland, therefore determining the genomic targets of RA in this gland will be an important future study; RA has been shown to regulate over 500 genes in other tissues, both directly and indirectly [53].

Increasing evidence is emerging for a role of RA as a regulator of biological rhythms in the central nervous system, in both circadian and seasonal rhythms (reviewed in Ransom et al. [13]). RA has been reported to influence components of the circadian clock, through inhibition of CLOCK:BMAL [18, 19], and upregulation of *Per1* [19], which may be through direct transcriptional control via RAREs found on the gene promoter regions [14, 15]. In order for RA to be a regulator of circadian rhythms it should be subject to diurnal variations in activity. The present study demonstrates that it is synthesized and signals on a diurnal basis, while also providing evidence for a role for RA in the pineal gland, an integral component of the circadian timing system. This study therefore supports the growing evidence for a new role for RA as a regulator of circadian rhythms.

In conclusion, this study demonstrates the presence of a new rhythmic hormonal signaling system in the rat pineal gland which uniquely is under both endogenous circadian and external light/dark cycle control. RA is a potent signaling molecule owing to its various genomic and non-genomic activities, therefore rhythmic RA synthesis and signaling could play an important modulatory role in the molecular rhythms in this gland, such as that of MAPK activation. The rapid effect of RA on ERK1/2 phosphorylation demonstrates that RA has the ability to signal rapidly in a system where precise temporal control is essential for the regulation of circadian rhythms. The pineal gland has an essential role in chronobiology as it is responsible for the conversion of time into a hormonal signal. It is therefore important to understand the molecular mechanisms underlying rhythmic pineal gland function which contribute to the precise control of the circadian rhythm in melatonin production.
